# Severe liver dysfunction complicating course of COVID-19 in the critically ill: multifactorial cause or direct viral effect?

**DOI:** 10.1186/s13613-021-00835-3

**Published:** 2021-03-15

**Authors:** Kevin Roedl, Dominik Jarczak, Andreas Drolz, Dominic Wichmann, Olaf Boenisch, Geraldine de Heer, Christoph Burdelski, Daniel Frings, Barbara Sensen, Axel Nierhaus, Marc Lütgehetmann, Stefan Kluge, Valentin Fuhrmann

**Affiliations:** 1 Department of Intensive Care Medicine, University Medical Center Hamburg-Eppendorf, Martinistraße 52, 20246 Hamburg, Germany; 2 Department of Internal Medicine I, University Medical Centre Hamburg-Eppendorf, Hamburg, Germany; 3 Institute of Medical Microbiology, Virology and Hygiene, University Medical Centre Hamburg-Eppendorf, Hamburg, Germany

**Keywords:** COVID-19, Hypoxic liver injury, Jaundice, Cholestatic liver disease

## Abstract

**Background:**

SARS-CoV-2 caused a pandemic and global threat for human health. Presence of liver injury was commonly reported in patients with coronavirus disease 2019 (COVID-19). However, reports on severe liver dysfunction (SLD) in critically ill with COVID-19 are lacking. We evaluated the occurrence, clinical characteristics and outcome of SLD in critically ill patients with COVID-19.

**Methods:**

Clinical course and laboratory was analyzed from all patients with confirmed COVID-19 admitted to ICU of the university hospital. SLD was defined as: bilirubin ≥ 2 mg/dl or elevation of aminotransferase levels (> 20-fold ULN).

**Results:**

72 critically ill patients were identified, 22 (31%) patients developed SLD. Presenting characteristics including age, gender, comorbidities as well as clinical presentation regarding COVID-19 overlapped substantially in both groups. Patients with SLD had more severe respiratory failure (paO_2_/FiO_2:_ 82 (58–114) vs. 117 (83–155); *p* < 0.05). Thus, required more frequently mechanical ventilation (95% vs. 64%; *p* < 0.01), rescue therapies (ECMO) (27% vs. 12%; *p* = 0.106), vasopressor (95% vs. 72%; *p* < 0.05) and renal replacement therapy (86% vs. 30%; *p* < 0.001). Severity of illness was significantly higher (SAPS II: 48 (39–52) vs. 40 (32–45); *p* < 0.01). Patients with SLD and without presented viremic during ICU stay in 68% and 34%, respectively (*p* = 0.002). Occurrence of SLD was independently associated with presence of viremia [OR 6.359; 95% CI 1.336–30.253; *p* < 0.05] and severity of illness (SAPS II) [OR 1.078; 95% CI 1.004–1.157; *p* < 0.05]. Mortality was high in patients with SLD compared to other patients (68% vs. 16%, *p* < 0.001). After adjustment for confounders, SLD was independently associated with mortality [HR3.347; 95% CI 1.401–7.999; *p* < 0.01].

**Conclusion:**

One-third of critically ill patients with COVID-19 suffer from SLD, which is associated with high mortality. Occurrence of viremia and severity of illness seem to contribute to occurrence of SLD and underline the multifactorial cause.

**Supplementary Information:**

The online version contains supplementary material available at 10.1186/s13613-021-00835-3.

## Background

The coronavirus disease 2019 (COVID-19) caused by novel severe acute respiratory syndrome coronavirus-2 (SARS-CoV-2) is responsible for a global threat for human health. Since its initial detection in Wuhan (China) in December 2019 COVID-19 spread and accounts for the ongoing pandemic with more than 30 million infections and 900.000 deaths [1, 2]. The disease is mainly characterized by mild flu-like symptoms or can be complicated by respiratory deterioration, potentially leading to acute respiratory distress syndrome (ARDS) and/or other organ failure [3–5]. A severe course of COVID-19 with need of intensive care unit (ICU) admission can be observed in up to 20% of hospitalized patients [6, 7]. Patients admitted to ICU suffer from high mortality [7, 8]. Several large studies reported clinical features and revealed that older age and underlying comorbidities increase the risk of unfavorable outcome [5, 6, 9–12]. Recent findings indicate that SARS-CoV-2 has an organotropism influencing the course of the disease and possibly aggravating pre-existing conditions [13].

However, it is unknown if liver damage in patients with COVID-19 is directly caused by viral infection of liver cells [14]. One autopsy study found liver cell alterations, possibly induced by COVID-19 [15], another recent report was able to identify SARS-CoV-2 in the cytoplasm of hepatocytes [16]. Different large studies reported liver injury during the course of COVID-19, often transient and returning to normal without special treatment [17–19]. However, its impact on outcome remains unclear and a wide variation in the incidence of liver injury was reported. Moreover, the use of anti-viral agents was associated with the occurrence of liver injury possibly explaining the large variation observed in different studies [18, 19]. Pre-existing liver diseases were reported in up to 11% of patients with COVID-19 [1, 5, 8, 10]. In patients with pre-existing liver cirrhosis and COVID-19, a deterioration of liver function and elevated mortality was observed [20].

In critically ill patients systemic inflammation resulting in cytokinemia as well as severe hypoxia because of pneumonia could contribute to liver damage. Liver injury and failure is a frequently observed type of organ failure in critically ill patients, and its occurrence is associated with increased morbidity and mortality [21–23]. Two major patterns can be clinically determined: cholestatic liver dysfunction (CLD) and hypoxic liver injury (HLI), which is also known as ischemic hepatitis or shock liver [24]. During the ICU stay up to 20% develop CLD and 10% suffer from HLI [21, 22, 25]. Especially, HLI frequently accompanies states of hypoxemia depletion in critically ill patients and could therefore be of special interest in COVID-19 [26]. However, occurrence and incidence of severe liver dysfunction (SLD) have not been reported systematically in critically ill patients with COVID-19.

Since data on SLD in critically ill patients with COVID-19 are lacking, we investigated occurrence, clinical characteristics and implications on outcome of critically ill patients with COVID-19 in a large tertiary care university hospital.

## Methods

### Study design, setting and ethics

Data of all adult patients with confirmed COVID-19 consecutively admitted to the Department of Intensive Care Medicine at the University Medical Centre Hamburg-Eppendorf (Germany) between March and July 2020 were analyzed. The department constitutes of 12 intensive care units (ICU) and cares for all critically ill adult patients of the hospital with a total capacity of 142 beds. The Ethics Committee of the Hamburg Chamber of Physicians was informed about the study (No.: WF-142/20). Due to the retrospective nature of the study, the need for informed consent was waived.

### Inclusion and exclusion criteria

All consecutive adult patients (≥ 18 years) with confirmed COVID-19 admitted to the ICU were included in the study. Confirmed COVID-19 was defined as at least one positive result on reverse transcriptase-polymerase chain reaction (rt-PCR) obtained from naso-pharyngeal swabs and/or bronchial secretions or blood. All patients < 18 years of age, or patients staying on the ICU at the end of the study were excluded.

### Data collection and virological diagnostics

Data were collected through electronical patient data management system (PDMS, Integrated Care Manager® (ICM), Version 9.1—Draeger Medical, Luebeck, Germany). The extracted data included age, gender, comorbidities, admission diagnosis, length of ICU stay, outcome, treatment modalities and organ support (mechanical ventilation, vasopressor, renal replacement therapy, blood transfusions, antibiotics, antivirals, etc.) and laboratory parameters. Routine laboratory assessment, including bilirubin, alanine aminotransferase and aspartate aminotransferase was performed daily. Respiratory samples (tracheal aspirates or throat swabs (eSwab, copan Italy)) as well as EDTA plasma and serum were obtained for surveillance as part of clinical routine. SARS-CoV-2 RNA was detected by real-time RT-PCR [27] using the Roche Cobas 6800 system. Viral RNA was quantified (from EDTA plasma and respiratory material) using in vitro transcribed SARS-CoV-2 RNA as standard as described previously [28].

### Study definitions and patient management

Severity of illness was evaluated by Sequential Organ Failure Assessment (SOFA) [29] and Simplified Acute Physiology (SAPS II) [30] Score on admission. Charlson Comorbidity Index (CCI) [31] was calculated in all patients. Sepsis and septic shock were defined according to the 2016 Third International Consensus Definition for Sepsis and Septic Shock [32]. Acute respiratory distress syndrome (ARDS) was defined according to the Berlin definition [33].

Severe liver dysfunction (SLD) was defined as occurrence hypoxic liver injury (HLI) and/or jaundice (defined as total bilirubin ≥ 2 mg/dl) [22]. HLI was diagnosed according to well-established criteria: (a) setting of cardiac, circulatory or respiratory failure; (b) dramatic but transient elevation of aminotransferase levels to at least 20-fold the upper limit of normal; (c) exclusion of other putative causes of liver cell necrosis (viral-/drug-induced hepatitis) [26]. Haemolysis, as cause of hyperbilirubinemia, was excluded by reviewing lactate dehydrogenase, haptoglobin and non-conjugated bilirubin levels. Pre-existing liver disease was excluded by using a combination of clinical, laboratory and radiological findings performed within routine clinical care. All patient charts were reviewed for presence of increased alcohol intake. Patient and ICU management was performed following national and international recommendations, including prone positioning in severe ARDS, and restrictive fluid management following the initial resuscitation period. Vasopressor support was initiated to obtain a mean arterial pressure (MAP) above 65 mmHg using norepinephrine [34, 35]. Viremia was defined as detection of viral RNA in blood and quantified as > 1000 copies/ml.

### Statistical analysis

Data are presented as absolute numbers and relative frequency or median and with interquartile range (IQR). Categorial variables were compared via Chi-square analysis or Fisher’s exact test, as appropriate. Continuous variables were compared via Mann–Whitney U test. We clinically assessed factors associated with the occurrence of SLD and mortality. We used multivariable logistic regression with SLD as the dependent variable and clinical variables as covariables. We used a multivariable Cox proportional hazards model to estimate the effect of SLD on ICU survival.

In both models we used a stepwise backward elimination approach that gradually reduces the initial model; variables that caused a change in estimates > 10%/statistically significant remained in the model). Statistical analysis was conducted using IBM SPSS Statistics Version 24.0 (IBM Corp., Armonk, NY). Generally, a p-value < 0.05 was considered statistically significant.

The study was prepared in accordance with the STROBE (STrengthening the Reporting of OBservational studies in Epidemiology) recommendations.

## Results

### Study population

**Fig. 1:**
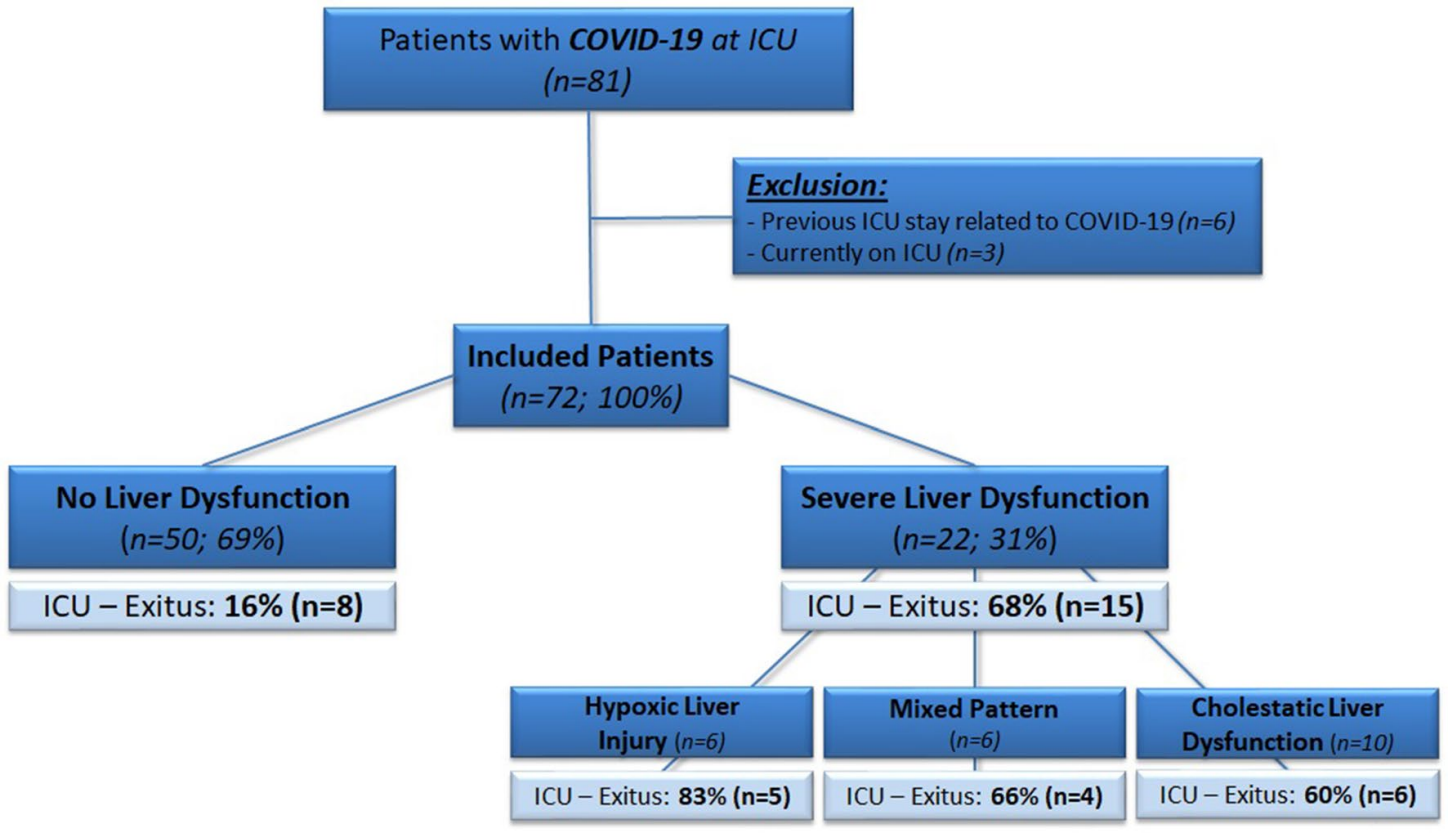
Flowchart of the study

During the study period (March 1^st^, 2020 to June 30^th^, 2020) a total number of 81 critically ill COVID-19 patients was treated at our department. After exclusion of patients currently staying in the ICU (*n* = 3) and patients with previous ICU stay related to COVID-19 (*n* = 6), we could include 72 patients in the final analysis (Fig. 1).

### Occurrence of severe liver dysfunction during intensive care unit stay

Of 72 patients, 22 (31%) developed severe liver dysfunction (SLD) during the ICU stay. The median time from ICU admission to SLD was 6 (4–11.3) days. We observed occurrence of a mixed injury pattern in 27% (*n* = 6); hypoxic liver injury or cholestatic liver dysfunction alone were observed in 27% (*n* = 6) and 45% (*n* = 10) patients, respectively.

### Clinical characteristics of patients on admission

Detailed characteristics on baseline are shown in Table 1. Demographic characteristics (age, gender and BMI) were similar in patients with and without SLD. Twenty-three (32%) patients presented with a BMI > 30 kg/m^2^, 39% (*n* = 9) suffered from SLD. Median time of symptom onset to ICU admission was 6.9 (2–13.8) days. Leading symptoms were shortness of breath (49%, *n* = 35), fever (47%, *n* = 34) and cough (40%, *n* = 29). Occurrence of symptoms was comparable in both groups. Median Charlson Comorbidity Index (CCI) was 2(1–3) points. Leading comorbidities were arterial hypertension, diabetes mellitus (type II) and chronic lung disease in 49% (*n* = 35), 32% (*n* = 23) and 14% (*n* = 10), respectively. No pre-existing liver disease was observed. Respiratory support was frequent on admission; 35% (*n* = 25) were mechanically ventilated and 13% (*n* = 9) received high-flow nasal cannula (HFNC) therapy. The median PaO_2_/FiO_2_ ratio (Horowitz Index) was 112 (84–168) on admission. Respiratory support as well as vital functions, including use of vasopressors, on admission was comparable in both groups. On admission higher median liver enzyme levels were observed; AST (56 vs. 43 U/l; *p* < 0.05) and ALT (44 vs. 31 U/l; *p* = 0.271) for patients with and without SLD, respectively. Further, median total bilirubin levels were significantly higher in patients with SLD (0.8 vs. 0.5 mg/dl; *p* < 0.01) on admission.

**Table 1:** Baseline characteristics of the study population

Variables	All (*n* = 72)	No liver dysfunction (*n* = 50)	Severe liver dysfunction (*n* = 22)	p-value
Age (years)	63 (54–73)	64 (55–73)	62 (51–73)	0.937
Males	51 (71)	34 (68)	17 (77)	0.308
BMI (kg/m^2^)	27.3 (24.8–31.7)	27.3 (24.9–30.8)	27.3 (24.3–33.1)	0.921
COVID-19 characteristics				
Symptoms to ICU (days)	6.9 (2–13.8)	6.7 (2.4–14.2)	6.5 (1.6–11.4)	0.476
Symptoms—before ICU				
Cough	29 (40)	20 (40)	9 (41)	0.783
Productive cough	8 (11)	4 (8)	4 (18)	0.214
Fever	34 (47)	23 (46)	11 (50)	0.581
Shortness of breath	35 (49)	26 (52)	9 (41)	0.579
Fatigue	15 (21)	10 (20)	5 (23)	0.748
Comorbidities				
Charlson comorb. index, pts	2 (1–3)	2 (1–3)	2 (1–3)	0.480
Arterial hypertension (*n, %)*	35 (49)	26 (52)	9 (41)	0.245
Pre-existing medication				
ACE inhibitor	15 (21)	12 (24)	3 (14)	0.255
Angiotensin receptor blocker	8 (11)	4 (8)	4 (18)	0.189
Chronic kidney disease	8 (11)	6 (12)	2 (9)	0.524
Coronary heart disease	8 (11)	6 (12)	2 (9)	0.524
Congestive heart failure	2 (3)	1 (2)	1 (5)	0.527
Diabetes mellitus	23 (32)	18 (36)	5 (23)	0.161
Chronic lung disease	10 (14)	8 (16)	2 (9)	0.341
Vital functions—admission				
Body temperature (C°)	37.3 (36.6–38.1)	37.4 (36.7–38.1)	37.3 (36.5–37.8)	0.482
Heart rate (beats/minute)	92 (81–108)	91 (81–108)	96 (80–107)	0.531
Mean arterial pressure (mmHg)	83 (72–100)	82 (74–103)	84 (71–90)	0.337
Vasopressor use	28 (39)	20 (40)	8 (36)	0.466
Respiratory support—admission				
paO_2_/FiO_2_	112 (84–168)	107 (83–168)	121 (102–160)	0.570
Invasive mechanical ventilation	25 (35)	19 (38)	6 (27)	0.271
Non-invasive ventilation	0 (0)	0 (0)	0 (0)	-
High-flow nasal cannula	9 (13)	4 (8)	5 (23)	0.091
Laboratory results—admission				
AST (U/l)	48 (30–75.8)	43 (29–70)	56 (42–78)	**0.035**
ALT (U/l)	34 (22–59)	31 (22–57)	44 (44–76)	0.271
Bilirubin (mg/dl)	0.6 (0.4–0.8)	0.5 (0.4–0.7)	0.8 (0.5–1.4)	**0.002**
Outcome				
Duration ICU stay (days)	13 (8.2–24.1)	12.4 (5.9–21.6)	16.8 (11.3–25.2)	0.285
Died in ICU	23 (32)	8 (16)	15 (68)	** < 0.001**

Data are expressed as n (%) or median (interquartile range)

*kg* kilogram, *m* meter, *ICU* intensive care unit, *BMI* body mass index, *pts* points, *AST* aspartate aminotransferase, *ALT* alanine aminotransferase; mg, milligram;

### ICU characteristics of patients with and without severe liver injury

Table 2 shows detailed characteristics on the ICU course and treatment modalities. All patients were admitted to the ICU due to respiratory deterioration. Patients with SLD had higher median SAPS II (SLD: 48 vs. without SLD: 40 points; *p* = 0.006) and SOFA score (7 vs. 6; *p* = 0.938) on admission, representing severity of illness. Median SOFA score 24 hrs after ICU admission was higher in patients with SLD (10 vs. 7; *p* = 0.075). ARDS was significantly more frequent in patients with (91%, *n* = 20) than in patients without SLD (60%, *n* = 30). Severe ARDS was more common in patients with SLD. Overall 74% (*n* = 53) patients needed invasive mechanical ventilation (MV) (95% vs. 64%; *p* = 0.004). The median duration of MV was 12.5 (7.3–25.8) days. Overall HFNC therapy, as well as non-invasive ventilation (NIV) was used in 35% (*n* = 25) and 10% (*n* = 7) patients, respectively. The median paO_2_/FiO_2_ ratio (Horowitz Index) was significantly lower in patients suffering from SLD (*p* = 0.030). Due to severe ARDS accompanied by life-threatening hypoxia, veno-venous extracorporeal membrane oxygenation (ECMO) was established in 12 patients. ARDS management included prone positioning (*n* = 41), glucocorticoid therapy (*n* = 26), inhaled vasodilatory treatment (*n* = 20) and neuromuscular blockade (*n* = 18). All ARDS interventions, except inhaled vasodilatory treatment, were significantly more commonly used in patients with SLD. Overall, 79% of patients received vasopressor therapy (95% vs. 72%; *p* = 0.027) and renal replacement therapy (RRT) was initiated in 47% of the cohort (86% vs. 30%; *p* < 0.001).

**Table 2:** ICU characteristics of patients with and without severe liver dysfunction

Variables	All (n = 72)	No liver dysfunction (*n* = 50)	Severe liver dysfunction (*n* = 22)	*p*-value
Disease severity				
SAPS II—admission (pts.)	41 (35–48.8)	40 (32–45)	48 (39–52)	**0.006**
SOFA—admission (pts.)	6 (3–11)	6 (3–11)	7 (3–9)	0.938
SOFA—24 h (pts.)	8 (4–13)	7 (3–11)	10 (7–15)	0.075
Respiratory support				
Invasive MV	53 (74)	32 (64)	21 (95)	**0.004**
Duration of MV (days)	12.5 (7.3–25.8)	13 (6–27)	12 (8–25)	0.965
Non-invasive ventilation	7 (10)	3 (6)	4 (18)	0.182
High-flow nasal cannula	25 (35)	16 (32)	9 (41)	0.454
ECMO	12 (17)	6 (12)	6 (27)	0.106
Worst paO_2_/FiO_2_	110 (71–151)	117 (83–155)	82 (58–114)	**0.033**
ARDS				
No ARDS	22 (31)	20 (40)	2 (9)	**0.007**
Mild	3 (4)	2 (4)	1 (5)	0.651
Moderate	19 (26)	13 (26)	6 (27)	0.258
Severe	28 (39)	15 (30)	13 (59)	0.225
ARDS—management				
Prone positioning	41 (57)	25 (50)	16 (73)	**0.036**
Neuromuscular blockade	18 (25)	10 (20)	8 (36)	**0.001**
Inhaled vasodilator	20 (28)	8 (16)	12 (55)	0.111
Glucocorticoid therapy	26 (36)	12 (24)	14 (64)	**0.001**
Procedures/therapies				
Vasopressors	57 (79)	36 (72)	21 (95)	**0.027**
Renal replacement therapy	34 (47)	15 (30)	19 (86)	** < 0.001**
Therapeutic anticoagulation	33 (46)	19 (38)	14 (64)	0.166
Antibiotic therapy	69 (96)	48 (96)	21 (95)	0.493
Experimental therapy				
Lopinavir, ritonavir	8 (11)	5 (10)	3 (14)	0.187
Remdesivir	2 (3)	1 (2)	1 (5)	0.521
Tocilizumab	3 (4)	0 (0)	3 (14)	**0.026**
Plasma exchange	2 (3)	1 (2)	1 (5)	0.521
Immunoglobulins	2 (3)	2 (4)	0 (0)	0.479
Laboratory results				
AST—peak (U/l)	140 (63–326)	88 (50–147)	746 (279–4293)	** < 0.001**
ALT—peak (U/l)	79 (39–172)	59 (32–103)	348 (127–720)	** < 0.001**
Bilirubin—peak (mg/dl)	0.8 (0.6–1.8)	0.5 (0.4–0.7)	2.6 (1.7–4.5)	** < 0.001**
pH—nadir	7.27 (7.14–7.39)	7.3 (7.19–7.39)	7.19 (7.07–7.29)	**0.015**
Lactate—peak	2.5 (1.7–5)	1.9 (1.7–2.9)	6.4 (3.4–12.8)	** < 0.001**
INR—admission	1.1 (1–1.2)	1.1 (1–1.1)	1.1 (1.03–1.2)	0.082
Thrombocytes—admission	204 (116–284)	227 (140–300)	161 (60–238)	0.078
Ferritin—admission	1493 (911–3009)	1249 (768–2326)	2395 (1126–6253)	**0.030**
IL-6—admission	134 (56–412)	104 (54–243)	279 (92–975)	**0.016**
LDH—admission	446 (364–603)	424 (364–577)	566 (380–681)	0.245
Complications—ICU stay				
Pulmonary embolism	3 (4)	1 (2)	2 (9)	0.219
Deep vein thrombosis	11 (15)	7 (14)	4 (18)	0.448
Cardiac arrest	11 (15)	5 (10)	6 (27)	0.068
Septic shock	32 (44)	16 (32)	16 (73)	**0.001**

Data are expressed as n (%) or median (interquartile range)

*ICU *intensive care unit, *BMI* body mass index, *ECMO* extracorporeal membrane oxygenation, *ARDS* acute respiratory distress syndrome, *AST* aspartate aminotransferase, *ALT* alanine aminotransferase, *LDH* lactate dehydrogenase;

Eight patients received lopinavir–ritonavir as specific anti-viral treatment, 3 patients received tocilizumab, 2 patients received remdesivir. Hydroxychloroquine was not used in our cohort. Two patients were treated with intravenous immunoglobulins and 2 received therapeutic plasma exchange.

During ICU stay complications were frequent: 44% (*n* = 32) suffered from septic shock, 25% (*n* = 11) suffered from cardiac arrest and 25% (*n* = 11) had deep-vein thrombosis. Pulmonary embolism was observed in 4% (*n* = 3) and newly developed heart failure in 3% (*n* = 3) patients. Cause of SLD was presumably septic shock in combination with ARDS in 73% (*n* = 16), ARDS only in 18% (*n* = 4) and cardiogenic shock in 9% (*n* = 2).

### Laboratory course

Higher peak values of AST, ALT and bilirubin were observed in patients with SLD (all *p* < 0.001). Further, worst pH was significantly lower and highest lactate was significantly higher in patients with SLD. Ferritin and IL-6 on admission were significantly higher in patients with SLD. The median time from admission to first rise of aminotransferases and bilirubin above defined thresholds for SLD was 8 (4.8–19.8) and 5.5 (2.8–12.5) days, respectively. For further laboratory results, see Table 1 and Additional file 1: Table S1.

### Virologic findings

Additional file 1: Table S2 shows the virologic findings in critically ill patients with COVID-19. 57% (*n* = 60) of patients with available blood samples had detectable virus RNA on admission. Further, virus was detected in 86% of upper and 90% of lower respiratory specimen on admission. No statistical difference in viral load from blood and upper- or lower-respiratory tract samples on admission were observed in patients with and without SLD.

During the ICU stay, 64% of patients had detectable virus RNA in blood samples. Virus RNA could be detected significantly more frequent in patients with SLD (84% vs. 55%; *p* = 0.024). Of patients with detectable viral load (*n* = 44), 32 (73%) presented with viremia. Viremia was present in 15 (68%) patients with and 17 (34%) patients without SLD (*p* = 0.002). Peak viral loads detected in blood, upper- and lower-respiratory tract samples were significantly higher (for all specimen) in patients with SLD.

### Risk factors for severe liver dysfunction and mortality

Multivariate regression analysis identified SAPS II [OR 1.078, 95% CI (1.004–1.157); *p* = 0.036] and presence of viremia [OR 6.359, 95% CI (1.336–30.253); *p* = 0.021] as factors significantly associated with new onset of HLI (see Table 3). After adjustment for confounders, we observed that SLD [HR 3.347, 95% CI (1.401–7.999); *p* = 0.006] and SAPS II [HR 1.049, 95% CI (1.002–1.097); *p* = 0.037] were significantly associated with ICU mortality (see Additional file 1: Table S3a).

**Table 3:** Multivariable logistic regression for factors associated with occurrence of severe liver dysfunction

Logistic regression	Covariables	OR (95% CI)	*p* value
Step 1	Age^a^	0.986 (0.938–1.038)	0.614
	SAPS II	1.073 (0.993–1.160)	0.073
	Septic shock (yes vs. no)	2.139 (0.490–9.338)	0.311
	ARDS (yes vs. no)	2.499 (0.932–6.701)	0.055
	Viremia^b^ (yes vs. no)	5,778 (1.160–28.769)	0.032
Step 2	SAPS II	1.066 (0.993–1.146)	0.083
	Septic shock (yes vs. no)	2.313 (0.545–9.803)	0.255
	ARDS (yes vs. no)	1.979 (0.672–3.865)	0.097
	Viremia (yes vs. no)	5.581 (1.133–27.491)	0.034
Final model	SAPS II	1.078 (1.004–1.157)	**0.036**
	ARDS (yes vs. no)	1.764 (0.938–1.925)	0.118
	Viremia (yes vs. no)	6.359 (1.336–30.253)	**0.020**

*OR* odds ratio, *CI* confidence interval, *ARDS* acute respiratory distress syndrome, *SAPS* II Simplified Acute Physiology Score II

^a^Age on admission was transformed prior to logistic regression analysis (natural logarithm)

^b^Viremia was defined as detectable viral RNA in blood quantified > 1000 copies/ml

### Outcomes of critically ill patients with COVID-19

Overall, we observed an ICU mortality of 32% (*n* = 23) in the total cohort. In patients with SLD, we observed an ICU mortality of 68% (*n* = 15) compared to 16% (*n* = 8) without SLD (*p* < 0.001). According to different patterns of liver dysfunction, we observed an ICU mortality of 83%, 66% and 60% in patients with hypoxic liver injury, mixed injury pattern and cholestatic liver dysfunction, respectively. The median length of ICU stay was 13 (8.2–24.1) days; 16.8 (11.3–25.2) days with SLD and 12.4 (5.9–21.6) without SLD.

## Discussion

In this study of critically ill patients with COVID-19, we found that occurrence of SLD was frequent and was significantly associated with increased mortality. To date, occurrence of SLD and implications on outcome have not been described in a cohort of critically ill patients with COVID-19. This is the first study investigating occurrence, clinical characteristics and implications on outcome of SLD in a cohort of critically ill patients with COVID-19.

Hospitalized patients with COVID-19 mainly suffer from respiratory deterioration. Patients with COVID-19 admitted to ICU are prone to respiratory failure with need for initiation of non-invasive and/or invasive respiratory support. In its severest form patients can develop ARDS, accompanied by severe states of oxygen depletion. Although, COVID-19 primary effects the respiratory system recent reports indicate that SARS-CoV-2 has an organotropism [13]. However, hepatic involvement in COVID-19 can be multifactorial due to direct cytopathic effects, uncontrolled immune reaction, sepsis or drug induced [36]. Liver alterations were found in autopsies of patients with COVID-19 [15, 16], probably one reason for the susceptibility of the liver may be the high expression of ACE2 receptor in cholangiocytes [36]. However, it remains unclear if this is either a direct viral effect or a multifactorial incident.

Liver involvement of SARS-CoV-2 is not surprising and was shown in other outbreaks of highly pathogenic human coronavirus in form of the middle-east respiratory syndrome (MERS) and SARS-CoV-1 before. In both diseases liver damage was a common feature during illness [37]. Further, liver damage was associated with severity of disease [37, 38]. Interestingly, according SARS-CoV-1 liver injury was reported as late occurrence compared to recent reports of SARS-CoV-2 and MERS [37, 38]. One study in critically ill patients with MERS reported a very high number of liver injury, but the threshold used for diagnosis was rather low probably leading to an overestimation of liver injury [38]. Summarized, liver injury is commonly reported in different highly pathogenic human coronavirus outbreaks. Definition of liver injury varied largely explaining differences in occurrence. However, implications on outcome in critically ill patients remain unclear [14, 36, 37].

Different studies in hospitalized patients suffering from COVID-19 reported abnormal liver function and/or liver injury in 14–53% [36] of patients. Most studies reported on non-critically ill hospitalized patients. Further, different definitions of liver failure were used complicating comparability between studies. However, highest rates of liver injury were reported in cases with severe COVID-19 [1, 39]. In deceased patients with COVID-19, one study reported acute liver injury in 10% of cases [40]. In our study, we observed an incidence of SLD in 31%. The higher prevalence can be a consequence of several factors. First, we used the widely accepted and well-established diagnostic criteria of HLI and jaundice in our cohort [21, 23–25, 41]. Earlier reports used much lower thresholds for diagnosis, leading to quite high rates of liver injury without any detectable effect on outcome. Second, we only report on critically ill patients including 74% of patients receiving mechanical ventilation due to severe COVID-19. Patients with severe pneumonia are prone to hypoxemia, probably triggering liver injury. This would be in line with earlier reports showing SLD can be caused by severe hypoxemia due to respiratory failure [41]. Furthermore, SLD was significantly more frequently accompanied by development of ARDS. As a consequence, significantly more patients with SLD were mechanically ventilated and the worst Horowitz Index (PaO_2_/FiO_2_ ratio) was lower. Furthermore, other factors can lead to occurrence of SLD in mechanically ventilated patients. Several studies in patients with liver transplantation and mechanical ventilation described decreased liver outflow and portal vein flow, possibly contributing to development of SLD [42, 43]. However, the results are conflicting and the effect of prolonged mechanical ventilation on liver hemodynamics has not been described. We observed several different other clinical characteristics accompanied with occurrence of SLD. Use of vasopressor, indicating presence of cardiac failure was observed in almost all patients with SLD, which was recently described in two studies in patients with cardiogenic shock and cardiac arrest [44, 45]. Acute kidney injury and necessity of RRT was a common observation in patients with SLD and was earlier described as marker for poor outcome [46]. Severity of illness represented by SAPS II and SOFA on admission and during course of ICU stay was higher in patients developing SLD, which is in line with earlier studies [47]. Further, SAPS II [OR 1.078; 95% CI 1.004–1.157] was independently associated with occurrence of SLD which confirms previous studies [47].

In general, liver injury and failure is a frequently observed in critically ill patients [23]. Traditionally, HLI and CLD were regarded as late features in critical illness [24]. However, recent findings showed that they are found early in life-threatening illness on the ICU [23–25]. In our cohort, SLD developed within one week after admission, with a median of 6 (4–11.3) days. The occurrence of HLI or CLD is associated with increased morbidity and mortality [21–23]. In the medical ICU about 20% develop CLD and 10% suffer from HLI [21, 22, 25]. In our cohort, we observed a higher rate of SLD, mainly as a consequence of the severity of COVID-19 with pronounced respiratory failure accompanied by sepsis and cardiac failure. Of interest, about 23% of patients with SLD had reduced ejection fraction measured by echocardiography. Further, Septic shock preceded SLD in 56% of patients. Presumably, the cause of SLD was septic shock combined with ARDS in more than 70% of our patients. However, data on patients with severe respiratory failure and/or ARDS are lacking. Mortality rates in patients with SLD were significantly higher in patients suffering from SLD. However, even after splitting injury patterns in HLI or CLD alone and a mixed injury pattern observed mortality rates were generally comparable between groups. Of interest, at time of death 12 of 15 patients met criteria of SLD. Highest mortality rate was observed in patients presenting with HLI injury pattern. Recent data showed that HLI accompanied by CLD increases complications and mortality [22]. After adjusting for covariables SLD was the most indicative factor [HR 3.347 95% CI 1.401–7.999] for mortality in our cohort of critically ill patients with COVID-19. A recent study showed that viral load can serve as predictor for mortality in patients with COVID-19 [48]. Interestingly, presence of viremia [OR 6.359 95% CI 1.336–30.253] was the most important factor associated with occurrence of SLD in our cohort, but was not associated with mortality. However, we observed significantly higher viral load as well as presence of viremia (defined as detection of viral RNA > 1000 copies/ml) in patients with SLD. This finding is novel and direct causation of liver injury by viral effects could be one explanation. Viremia was not associated with mortality in our cohort; this could be explained by the rather small number of patients included. Nevertheless, occurrence of viremia in critically ill patients (especially in patients with SLD) could serve as an early risk marker and should prompt close surveillance of liver function. Different clinical patterns and complications, like hypo-/hyperglycemia, hyperammonemia, respiratory failure or acute kidney injury in patients with SLD are associated with increased mortality [21–24, 46, 47]. Due to occurrence of SLD within a dynamic process in patients with multi-organ failure patients are generally not eligible for liver transplantation. However, even though no specific treatment of SLD is available, prevention of complications is of central importance. All in all, our data clearly demonstrate that occurrence of SLD is a multifactorial event. Severity of illness as well as complications during ICU, and direct viral effects play an important role in development of SLD.

Although, the number comorbidities in our cohort were high, no patient suffered from pre-existing liver disease. Pre-existing liver diseases were reported in up to 11% of patients with COVID-19 [1, 5, 8, 10] and were associated with fast deterioration of liver function and elevated mortality [20]. Thirty-two per-cent of patients presented with a BMI > 30 kg/m^2^. Although, we did not observe differences in occurrence of SLD in our cohort patients with non-alcoholic fatty liver disease or non-alcoholic steatohepatitis are probably at risk for SLD. However, whether COVID-19 aggravates occurrence of SLD or not must be addressed in larger analyses of critically ill patients with COVID-19 and pre-existing liver disease.

This study has strengths and limitations. First, the sample size of our study is rather small. However, this is the first and most comprehensive study describing the occurrence of SLD in critically ill patients with COVID-19. Second, for SLD we used a combination of well-established definitions of HLI and CLD. Both entities can be caused by different underlying mechanism, therefore our definition has to be interpreted with caution when comparing or data to other studies. Third, for detection of pre-existing liver disease, we carefully reviewed patient charts and due to routine clinical care patients were screened for presence of liver disease. However, due to the retrospective character of the study, we cannot entirely exclude the presence of pre-exiting liver disease. Fourth, we show results of a center experienced in management of ARDS and liver failure, and results and conclusions may not be generally transferable to other settings with less experience. Fifth, direct viral effects of SARS-CoV-2 could not be further validated in this clinical study and should be addressed in future studies. Sixth, residual confounding from unmeasured covariables is a matter of concern and cannot be entirely excluded. Future larger studies should be conducted to confirm these results.

## Conclusion

In conclusion, our study could demonstrate that occurrence of SLD is a frequent observation in critically ill patients with COVID-19 and is associated with high mortality rates. Severity of illness as well as viremia seems to contribute to the occurrence of SLD and underline the multifactorial cause. Our findings highlight the significant contribution and impact on outcome of SLD in critically ill patients with COVID-19.

## Supplementary Information

**Additional file 1: Table S1.** Biomarkers stratified according no liver dysfunction and severe liver dysfunction. **Table S2.** Viral characteristics in patients with and without severe liver dysfunction. **Table S3a.** Cox regression model for factors associated with ICU mortality.

## Data Availability

The datasets supporting the conclusions of this article are included within the article.

## Abbreviations

ARDS: Acute respiratory distress syndrome
CCI: Charlson Comorbidity Index
CLD: Cholestatic liver dysfunction
COVID-19: Coronavirus disease 2019
ECMO: Extracorporeal membrane oxygenation
HFNC: High-flow nasal cannula
HLI: Hypoxic liver injury
ICU: Intensive care unit
IQR: Interquartile range
MAP: Mean arterial pressure
MV: Mechanical ventilation
NIV: Non-invasive ventilation
RRT: Renal replacement therapy
SARS-CoV-2: Severe acute respiratory syndrome coronavirus-2
SAPS II: Simplified Acute Physiologic Assessment Score
SOFA: Sequential Organ Failure Assessment
