# Supplementary material for: Severe liver dysfunction complicating course of COVID-19 in the critically ill: multifactorial cause or direct viral effect?
Source: Ann Intensive Care. 2021 Mar 15;11:44. doi: 10.1186/s13613-021-00835-3 (PMC7957439; doi:10.1186/s13613-021-00835-3)
Supplement: Supplementary file 1 — Additional file 1: Table S1. Biomarkers stratified according no liver dysfunction and severe liver dysfunction. Table S2. Viral characteristics in patients with and without severe liver dysfunction. Table S3a. Cox regression model for factors associated with ICU mortality. [file 13613_2021_835_MOESM1_ESM.docx]

Supplementary Table 1 – Biomarkers

| *Variables* | *All*  *(n = 72)* | *No liver dysfunction*  *(n = 50)* | *Severe liver dysfunction*  *(n = 22)* | *p*-value |
| --- | --- | --- | --- | --- |
| Laboratory results  Haemoglobin – admission  Haemoglobin – 24h  Leukocytes - admission  Leukocytes - 24h  Thrombocytes - admission  Thrombocytes - 24h  Creatinine - admission  Creatinine - 24h  PCT - admission  PCT - 24h  D-Dimer – admission  D-Dimer – 24h  CRP – admission  CRP – 24h  INR – admission  INR – 24h  IL-6 – admission  IL-6 – 24h  Ferritin - admission  Ferritin – 24h | 10.7 (8.8 – 12.7)  9.8 (8.6 – 11.8)  8.5 (4.4 – 12.3)  8.8 (5.1 13.3)  204 (116 – 284)  217 (149 – 277)  0.93 (0.69 – 2)  0.98 (0.73 – 1.78)  0.38 (0.20 – 1.09)  0.51 (0.18 – 2.17)  2.97 (1.49 – 8.7)  3.3 (1.5 – 8.7)  162 (82 – 276)  171 (86 – 288)  1.1 (1 – 1.2)  1.1 (1 – 1.2)  134 (56 – 412)  148 (60 – 423)  1493 (911 – 3009)  1485 (986 – 3109) | 10.9 (8.8 – 12.4)  10 (8.7 – 11.8)  8.5 (4.7 – 12.2)  8.6 (5.2 – 13.3)  227 (140 – 300)  237 (156 – 298)  0.92 (0.69 – 2.47)  0.97 (0.73 – 1.65)  0.33 (0.18 – 0.92)  0.5 (0.15 – 1.46)  2.95 (1-29 – 7.51)  2.3 (1.24 – 7.51)  134 (63 – 274)  138 (73 – 274)  1.1 (1 – 1.1)  1.1 (1 – 1.1)  104 (54 – 243)  99 (47 – 312)  1249 (768 – 2316)  1390 (982 – 2352) | 9.9 (8.5 – 12.9)  9.2 (8.3 – 11.4)  8.6 (2.8 – 13.2)  9.3 (5.1 – 12.4)  161 (60 – 238)  180 (50 – 236)  0.99 (0.73 – 1.78)  0.99 (0.73 – 1.78)  0.40 (0.27 – 2.07)  0.93 (0.33 – 7.74)  3.51 (1.96 – 20.34)  5.04 (2.55 – 14.33)  185 (147 – 292)  219 (166 – 306)  1.1 (1.0 – 1.2)  1.1 (1 – 1.3)  279 (93 – 975)  238 (139 – 679)  2395 (1126 – 6253)  2681 (1130 – 5770) | 0.612  0.447  0.854  0.827  0.078  0.096  0.951  0.922  0.183  0.076  0.273  **0.043**  **0.018**  0.087  0.082  **0.039**  **0.016**  **0.019**  **0.030**  0.058 |
| Blood gas analysis  paO_2_ – admission  paO_2_ – 24h  paCO_2_ – admission  paCO_2_ – 24h  pH – admission  pH – 24h  Lactate – admission  Lactate – 24h | 73 (64 – 90)  75 (62 – 81)  38 (33 – 45)  39 (35 – 47)  7.44 (7.35 – 7.49)  7.42 (7.36 – 7.48)  1.1 (0.8 – 1.4)  1.2 (0.8 – 1.8) | 75 (65 – 92)  77 (69 – 87)  38 (33 – 46)  39 (35 – 44)  7.44 (7.34 – 7.48)  7.41 (7.36 – 7.46)  0.9 (0.7 – 1.4)  1.1 (0.7 – 1.6) | 71 (62 – 78)  69 (61 – 77)  36 (32 – 45)  39 (35 – 49)  7.46 (7.38 – 7.5)  7.42 (7.32 – 7.47)  1.2 (0.9 – 2.3)  1.2 (1 – 2) | 0.497  **0.040**  0.619  0.551  0.143  0.648  **0.037**  0.150 |

***Data are expressed as n (%) or median (interquartile range)****Abbreviations:* PCT, procalcitonin; CRP, c-reactive protein;

Supplementary Table 2 – Viral Characteristics in patients with and without severe liver dysfunction

| *Variables* | *All* | *No liver dysfunction* | *Severe liver dysfunction* | *p*-value |
| --- | --- | --- | --- | --- |
| Place of viral detection  *Admission* Blood  Upper – Respiratory Tract  Lower – Respiratory Tract  *Overall*  Blood  Upper – Respiratory Tract  Lower – Respiratory Tract | 34/60 (57)  24/28 (86)  37/41 (90)  42/66 (64)  33/47 (70)  56/59 (95) | 21/41 (51)  17/21 (81)  25/29 (86)  26/47 (55)  24/37 (65)  37/40 (93) | 13/19 (68)  7/7 (100)  12/12 (100)  16/19 (84)  9/10 (90)  19/19 (100) | 0.166  0.292  0.235  **0.024**  0.122  0.304 |
| Quantification of viral load*  *Admission* Blood (copies/ml)  Upper – Respiratory Tract (copies/ml)  Lower – Respiratory Tract (copies/ml)  *Peak value*  Blood (copies/ml)  Upper – Respiratory Tract (copies/ml)  Lower – Respiratory Tract (copies/ml) | 2.15x10³ (7.98x10² - 6.45x10³)  2.03x10^5^ (5.97x10³ - 1.02x10^7^)  4.27x10^5^ (2.34x10^4^ - 8.99x10^6^)  3.5x10³ (1.19x10³ - 1.35x10^4^)  1.15x10^6^ (1.24x10^4^ - 3.98x10^7^)  3.33x10^6^ (7.32x10^4^ - 2.14x10^7^) | 1.53x10³ (7.74x10² - 4.26x10³)  4.12x10^4^ (4.27x10³ - 9.65x10^6^)  3.98x10^5^ (2.34x10^4^ - 4.43x10^6^)  1.82x10³ (8.68x10² - 4.44x10³)  2.03x10^5^ (4.51x10³ - 1.93x10^7^)  9.32x10^5^ (1.00x10^4^ - 1.13x10^7^) | 5.66x10³ (2.15x10³ - 1.42x10^4^)  8.68x10^5^ (4.37x10^5^ - 1.97x10^7^)  6.55x10^5^ (5.99x10^4^ - 1.36x10^7^)  1.09x10^4^ (3.56x10³ - 3.21x10^5^)  2.26x10^7^ (8.68x10^5^ - 2.70x10^8^)  1.11x10^7^ (7.02x10^5^ - 1.39x10^9^) | **0.060**  0.494  0.378  **0.008**  **0.040**  **0.028** |
| Viraemia^1^  Admission  During ICU | 24/34 (71)  32/42 (76) | 11/21 (52)  17/26 (65) | 13/13 (100)  15/16 (94) | 0.051  **0.002** |

***Data are expressed as n (%) or median (interquartile range), *Viral load: copies/ml; ^1^*** Viraemia as defined as detectable viral RNA in blood quantified > 1000 copies/ml

Supplementary Table 3a – Cox-Regression model for Factors associated with ICU-Mortality

| *Cox regression* | Covariables | HR (95% CI) | *p* value |
| --- | --- | --- | --- |
| *Step 1* | **Severe liver dysfunction** (yes vs. no)  **Renal replacement therapy** (yes vs. no)  **Septic shock** (yes vs. no)  **ARDS** (yes vs. no)  **Vasopressor** (yes vs. no)  **SAPS II**  **Viraemia**^1^ (yes vs. no) | 3.700 (0.946 – 14.467)  0.384 (0.055 – 2.669)  2.634 (0.728 – 9.528)  0.284 (0.024 – 3.268)  0.411 (0.150 – 1.128)  1.049 (0.998 – 1.102)  1.507 (0.411 – 5.519) | 0.060  0.333  0.139  0.312  0.083  0.057  0.535 |
| *Step 2* | **Severe liver dysfunction** (yes vs. no)  **Renal replacement therapy** (yes vs. no)  **Septic shock** (yes vs. no)  **ARDS** (yes vs. no)  **Vasopressor** (yes vs. no)  **SAPS II** | 4.830 (1.587 – 14.703)  0.349 (0.050 – 2.410)  2.782 (0.767 – 10.087)  0.285 (0.024 – 3.280)  0.325 (0.131 – 1.009)  1.045 (0.996 – 1.096) | 0.005  0.286  0.119  0.314  0.193  0.070 |
| *Step 3* | **Severe liver dysfunction** (yes vs. no)  **Renal replacement therapy** (yes vs. no)  **Septic shock** (yes vs. no)  **Vasopressor** (yes vs. no)  **SAPS II** | 4.729 (1.555 – 14.378)  0.281 (0.045 – 1.747)  2.647 (0.739 – 9.483)  0.129 (0.104 – 0.992)  1.047 (0.997 – 1.099) | 0.006  0.173  0.134  0.294  0.061 |
| *Step 4* | **Severe liver dysfunction** (yes vs. no)  **Renal replacement therapy** (yes vs. no)  **Septic shock** (yes vs. no)  **SAPS II** | 4.779 (1.568 – 14.562)  0.298 (0.046 – 1.903)  2.772 (0.760 – 10.113)  1.047 (0.997 – 1.099) | 0.005  0.200  0.122  0.061 |
| *Step 5* | **Severe liver dysfunction** (yes vs. no)  **Septic shock** (yes vs. no)  **SAPS II** | 3.349 (1.377 – 8.140)  1.888 (0.620 – 5.750)  1.041 (0.994 – 1.090) | 0.007  0.262  0.081 |
| *Final model* | **Severe liver dysfunction** (yes vs. no)  **SAPS II** | 3.347 (1.401 – 7.999)  1.049 (1.002 – 1.097) | **0.006**  **0.037** |

Abbreviations: HR, hazard ratio; CI, confidence interval; ARDS, acute respiratory distress syndrome; SAPS II, simplified acute physiology score II; *Age on admission was transformed prior to logistic regression analysis (natural logarithm). ^1^ Viraemia as defined as detectable viral RNA in blood quantified > 1000 copies/ml
